# The surge of HIV in Pakistan: an epidemic or a progressive transmission in denial? An editorial

**DOI:** 10.1097/MS9.0000000000000172

**Published:** 2023-02-17

**Authors:** Abdul Moiz Khan

**Affiliations:** Ayub Medical College, Abbottabad, Pakistan

*Dear Editor*,

The current surge of diagnosed HIV cases in Pakistan has taken the general population by shock. Less because of its severity and more because of the stigmas revolving around it. The *Lancet’s* article ‘Pakistan’s growing HIV epidemic’ sheds light on the recently diagnosed 8262 HIV cases from January to September 2022^1^. However, it is a matter of interest if it really is an epidemic. The Centers for Disease Control and Prevention define an epidemic as ‘an unexpected sudden increase in the number of disease cases in a specific geographical area’^2^. In a country like Pakistan, this surge in HIV cases is less of an ‘unexpected’ rise and more of a predicted yet highly overlooked problem. According to the National AIDS Control Program’s official data, there are an estimated 0.2 million cases of HIV, yet only 53 718 are registered^3^. It is an injustice to the statistics if the government, already informed about the huge number of contagious unregistered patients, fails to adopt policies to contain the further spread. The prevalence of HIV is not an overnight surge but has been persistently increasing over the past three decades^4^.

Lack of knowledge about this disease has accumulated various superstitions and taboos around the topic, which greatly hampers efforts to address the management of HIV^5^. HIV transmits via the exchange of various body fluids from an infected patient, such as blood, milk, and semen. It may also have a vertical spread from the mother to the fetus^6^. But the population in Pakistan is unaware. According to a report, only 13% of the general population can name the three modes of transmission of AIDS, while 50% of the population has never heard of AIDS^7^. The general population considers HIV to spread only through immoral sexual practices. However, Pakistanis tend to believe that HIV transmission through illicit sexual activity cannot be a problem in the Muslim world^8^, thus narrowing the scope of preventive measures that can be adopted for fighting it. This grants other modes of transmission the liberty to spread disease. A big reason for the spread of HIV is blood contact in medical practices, barber shops, etc. However, they continue to be a neglected threat to society. Many cases of the spread of AIDS in Pakistan are due to the use of contaminated needles due to a lack of awareness^9^.

It is a fact, there is no riddance from AIDS without public health interventions changing the narrative of the community. Contagious diseases demand efforts for public awareness and prevention as much as treatment, if not more. Without proper knowledge and preventive measures, the dangers of HIV are not going away anytime soon. The threat has always been there. It was just going unaddressed. HIV will continue to spread in Pakistan unless solid measures are taken by national policymakers. It is high time we realize that merely treating the disease is not enough. Educating the masses, countering the stigmas, and changing perceptions are very crucial for us to win this fight. The government must take strict action to deal with it and not turn a blind eye to this progressive transmission.

## Ethical approval

NA.

## Consent

Not required.

## Sources of funding

None.

## Author contribution

Abdul Moiz Khan did every task.

## Conflicts of interest disclosure

The authors declare that they have no financial conflict of interest with regard to the content of this report.

## Research registration unique identifying number (UIN)

None.

## Guarantor

Abdul Moiz Khan.

## Provenance and peer review

Not commissioned, externally peer reviewed.
